# Interpretable hybrid deep learning features of pectoralis on low-dose chest CT for detecting type 2 diabetes

**DOI:** 10.3389/fendo.2026.1926903

**Published:** 2026-09-16

**Authors:** Yuning Guo, Song Wang, Jiaxun Lai, Xueyan Liu, Shunyi Lyu, Huimei Chao, Dewei Kong, Xifu Wang, Qiufeng Zhao

**Affiliations:** 1 Department of Radiology, Longhua Hospital, Shanghai University of Traditional Chinese Medicine, Shanghai, China; 2 Department of Rehabilitation Medicine, Shuguang Hospital Affiliated to Shanghai University of Traditional Chinese Medicine, Shanghai, China; 3 Department of Radiology, Shanghai General Hospital, Shanghai, China

**Keywords:** deep learning, explainable artificial intelligence, low-dose chest computed tomography, pectoralis, type 2 diabetes

## Abstract

**Objective:**

To develop an explainable hybrid deep learning features framework using low-dose chest CT (LDCT)-derived pectoralis features for opportunistic type 2 diabetes mellitus (T2DM) opportunistic screening, evaluating its incremental value over conventional biomarkers.

**Methods:**

This multi-center study analyzed LDCT images from 1,209 individuals. Following automated MedSAM segmentation, conventional metrics (e.g., Mean_HU, SMI) and deep learning (DL) features (ResNet152) were extracted. To prevent data leakage, PCA, LASSO selection, and ExtraTrees model optimization were strictly confined to the training set. A hybrid (COM) model integrating both feature types was compared against a baseline Muscle model. SHapley Additive exPlanations (SHAP) provided clinical interpretability.

**Results:**

The COM model achieved an area under the curve (AUC) of 0.830 (training), 0.759 (internal validation), and 0.746 (external validation). While performing comparably to the DL model without significant difference across validation cohorts (DeLong p > 0.05), it significantly outperformed the baseline Muscle model (external AUC: 0.515; DeLong test, p < 0.001). SHAP analysis confirmed that lower Mean_HU correlated with higher T2DM risk, while DL signatures provided robust, independent diagnostic value.

**Conclusion:**

This explainable hybrid model and DL model capture sub-visual pectoralis alterations, offering significant incremental diagnostic value over simple clinical and imaging metrics. They serves as an efficient, non-invasive tool for opportunistic T2DM screening during routine LDCT.

## 1 Introduction

The global prevalence of diabetes mellitus (DM) has reached alarming levels, with over 537 million adults affected worldwide in 2021, a number projected to rise to 783.2 million (12.2%) by 2045 (1). Beyond its increasing prevalence, type 2 diabetes mellitus (T2DM) is associated with a wide spectrum of complications, including cardiovascular, renal, and neurological disorders. These complications significantly impact patients’ quality of life and impose a significant economic burden on healthcare systems (2). Early diagnosis of T2DM is therefore essential to reduce the risk of complications, improve patient outcomes, and optimize healthcare resource allocation.Skeletal muscle, one of the body’s largest and highly insulin-sensitive organs, plays a critical role in glucose uptake and metabolism. T2DM is characterized not only by abnormal skeletal muscle metabolism but also by a reduction in muscle mass and strength indicators (e.g., grip strength). A decline in muscle mass can hinder glucose processing, thereby exacerbating disturbances in blood sugar metabolism (3). This characteristic allows skeletal muscle to serve as both an insulin-sensitive tissue and a significant predictor of diabetes risk. Relevant reviews indicate that sarcopenia, defined by a progressive loss of muscle mass, strength, and function, establishes a vicious cycle with T2DM, further worsening muscle decline in affected individuals (4). Consequently, skeletal muscle mass and metabolic health can serve as dynamic biomarkers for monitoring T2DM risk and progression. Recent advances in imaging have provided new opportunities to leverage this relationship for early detection (5). Ultrasound studies have revealed characteristic hyperechoic patterns in the deltoid muscle of patients with T2DM and prediabetes, which demonstrate high predictive value for T2DM (4, 6). Similarly, artificial intelligence (AI) models applied to digital breast tomosynthesis (DBT) images have achieved high accuracy (92%) in detecting diabetes in women by analyzing pectoralis muscle characteristics (7). These findings highlight the potential of AI-driven image features- such as shape, volume, and area- for T2DM detection.

The pectoralis muscle, critical for motor function, has also emerged as a valuable imaging biomarker for disease detection and health screening. Its imaging characteristics, particularly cross-sectional area and density derived from CT, have been validated as reliable indicators for diagnosing sarcopenia (8). Notably, Chinese cancer statistics show a sharp increase in lung cancer incidence and mortality after age 45, leading to recommendations for initiating lung cancer screening at this age (9, 10). In parallel, the American Diabetes Association (ADA) advises commencing diabetes screening at age 45 (11). These overlapping screening thresholds suggest an opportunity to exploit low-dose chest CT (LDCT), widely used in lung cancer screening, for additional diagnostic insights.

Based on this rationale, we propose a novel hybrid deep learning (DL) and machine learning (ML) framework that utilizes pectoralis imaging features extracted from routine LDCT scans to detect T2DM. Crucially, to bridge the gap between “black-box” deep learning and clinical practice, we incorporate SHapley Additive exPlanations (SHAP) into our framework. This analysis aims to provide clinical transparency and validate the biological grounding of the detective features, ensuring the model aligns with established physiological paradigms. This approach aims to provide dual diagnostic benefits—enabling both lung cancer screening and T2DM concurrent triaging—within a single imaging modality.

## 2 Materials and methods

### 2.1 Patients

We retrospectively reviewed electronic medical records from the health check-up centers at Center I (n=1158) between January 2021 and March 2023 and at Center II (n=152) between December 2022 and March 2023 (**Figure 1**). The inclusion criteria were as follows: (a) individuals who underwent LDCT; (b) HbA1c testing was performed within one week prior to or after LDCT; and (c) for individuals with HbA1c between 5.7% and 6.5%, an Oral Glucose Tolerance Test (OGTT) was performed. Exclusion criteria were as follows: type 1 diabetes (n=31), poor-quality CT images (n=48), prior thoracic or breast surgery (n=11), and history of chest radiotherapy (n=10), chronic kidney disease, liver disease, neuromuscular diseases, long-term use of steroids(n=1). Participants were classified as having T2DM if they met any of the following criteria (1): a documented prior clinical diagnosis of T2DM (2); HbA1c ≥ 6.5% (3); fasting plasma glucose ≥ 7.0 mmol/L; or (4) a 2-hour post-load blood glucose ≥ 11.1 mmol/L during the OGTT. In total, 1069 participants from Center I (327 T2DM and 742 non-T2DM cases), and 140 participants from Center II (45 T2DM and 95 non-T2DM cases), were included in the final cohort. Participants from Center I (n=1069) were used for model construction, randomly divided into a training cohort (n=855) and an internal validation cohort (n=214). Additionally, an external validation cohort (n=140) was collected from Center II.

**Figure 1:**
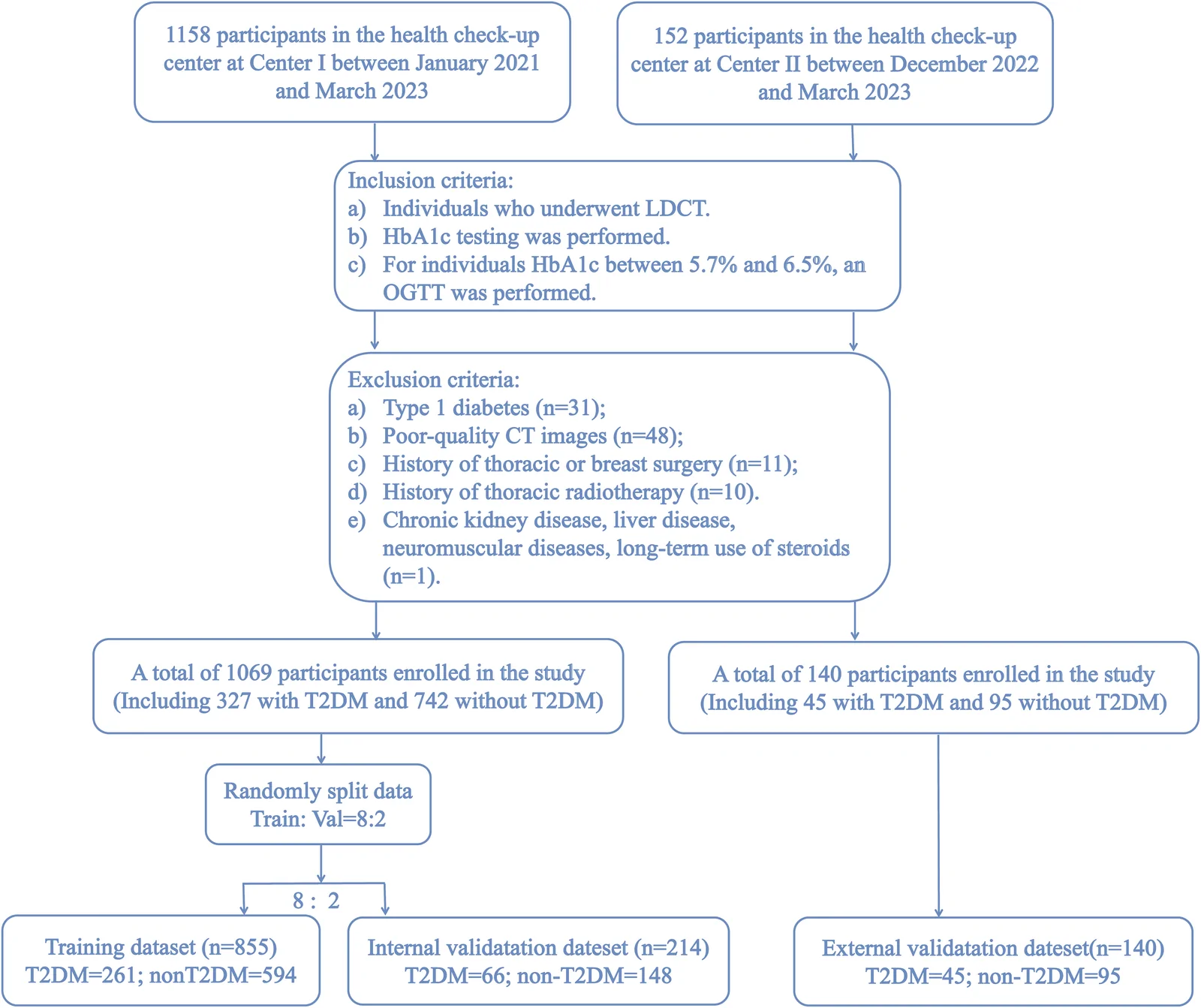
Flowchart of patient selection. Non-T2DM non-type 2 diabetes mellitus, OGTT oral glucose tolerance test, T2DM type 2 diabetes mellitus, HbA1c glycated hemoglobin A1c.

### 2.2 CT acquisition

LDCT scans were performed at both centers using different CT scanners. At Center I, a 64-slice spiral CT scanner (Optima CT620; GE Healthcare, USA) was used with the following parameters: 100 kV, 40 mA, 512 × 512 matrix, slice thickness and interval of 1.25 mm. At Center II, a 256-slice spiral CT scanner (Brilliance iCT; Philips, Netherlands) was employed with similar parameters except for a slice thickness and interval of 1mm.

### 2.3 ROI segmentation

#### 2.3.1 Images for segmentation and preprocessing

Accurate segmentation of the pectoralis muscle was essential for subsequent analysis. The region of interest (ROI) was defined on the axial CT slice showing the maximum cross-sectional area of the pectoralis (12). To minimize variability due to different scanners and protocols, all CT images were resampled to an isotropic voxel size of 1 × 1 × 1 mm³. Reconstructions were performed using a standardized soft tissue window (width: 400, level: 40).

Manual segmentation of the pectoral was independently performed by two radiologists (radiologists A and B, with 3 and 10 years of experience in chest CT diagnosis, respectively), both blinded to participants’ T2DM status. Radiologist A conducted the initial delineation, which was reviewed and refined by radiologist B. Segmentation was performed using ITK-SNAP software (version 3.6.0).

#### 2.3.2.Semi-automated segmentation model and postprocessing

To enable automated segmentation, we fine-tuned the MedSAM model for pectoralis muscle delineation. The fine-tuning strategy involved freezing the image encoder and bounding box prompt encoder while retraining the mask decoder. A total of 300 annotated CT images were used to fine-tune the model, and performance was evaluated on the internal dataset. The segmentation process (**Figure 2**) included: (i) manual placement of a bounding box to localize the pectoralis, (ii) generation of an initial segmentation mask using the fine-tuned MedSAM model, and (iii) manual correction of results by radiologist B. This semi-automated approach significantly reduced the time required per slice (from approximately 3 minutes for full manual contouring to less than 15 seconds). To evaluate reproducibility and inter-observer variability, bounding box placement and manual corrections were independently performed by two radiologists on a random subset of 100 cases. Inter-observer agreement was quantified using the intra-class correlation coefficient (ICC>0.85) for the extracted muscle area. Furthermore, to ensure broad generalizability, the segmentation performance (Dice and Jaccard index [IoU]) was evaluated not only on the internal dataset but also separately on the external validation cohort.

**Figure 2:**
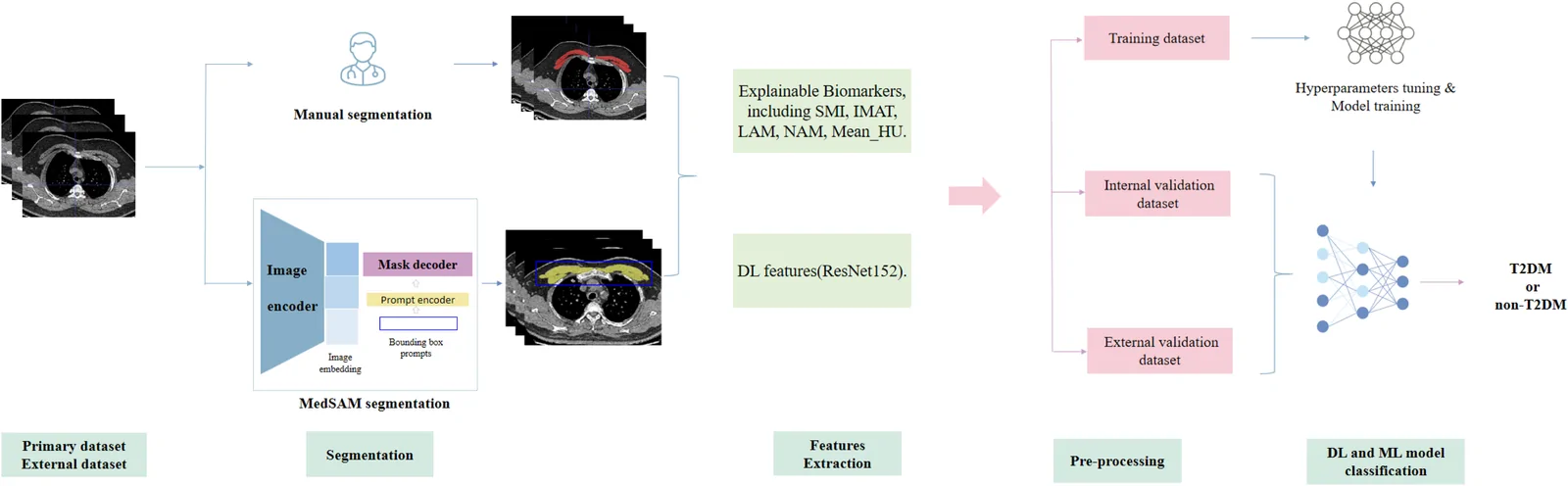
The flowchart illustrates the DL and ML models used for lesion classification after the preprocessing and segmentation of the chest muscle CT image. T2DM type 2 diabetes mellitus, Non-T2DM non-type 2 diabetes mellitus.

### 2.4 Simple and explainable biomarkers models

Cross-sectional pectoral muscle area was determined based on CT data of the pectoral muscles above the aortic arch, and the skeletal muscle area, skeletal muscle index (SMI), mean HU and intermuscular fat tissue (IMAT) were calculated. Low-attenuation muscle (LAM) and normal-attenuation muscle (NAM) were quantified using thresholds of –29 to +29 HU and +30 to +150 HU, respectively (13).

### 2.5 DL model

#### 2.5.1 DL feature extraction and hybrid feature selection

Deep learning features were extracted from the defined ROIs (bounding boxes around the largest pectoralis cross-section above the aortic arch) using a pre-trained DL architecture, ResNet152, initialized with ImageNet weights. The ResNet152 architecture was adapted to develop a convolutional neural network (CNN) for extracting DL features. The parameters of these CNN models were pretrained using all the maximum ROI images to generate DL features. Subsequently, the pretrained CNN models were used to extract DL features for each maximum ROI image, with the output from the penultimate layer serving as the DL features. To match the 3-channel RGB input requirement of the ImageNet-pretrained ResNet152, the single-channel gray-scale CT ROI images were duplicated across three channels. The cropped ROI images were resized to 224×224 pixels using bilinear interpolation. No spatial augmentation was applied during extraction to preserve the original anatomical proportions. Features were extracted from the final average pooling layer (avg_pool) of the ResNet152 network, yielding an initial high-dimensional vector of 1024 DL features per patient. Principal component analysis (PCA) was first applied to the training set, reducing the feature dimensions to 64 principal components. The same PCA transformation was then applied to the validation and test sets to ensure consistency in dimensionality reduction across all datasets. These PCA-reduced features were merged with conventional explainable biomarkers (e.g., SMI, LAM, NAM, and Mean_HU). Before feature selection, standardization (using the z-score method) and correlation filtering (Pearson r ≥ 0.9) were derived exclusively from the training set, and these parameters were subsequently applied to the validation datasets. Following this, the least absolute shrinkage and selection operator (LASSO) algorithm was applied. The optimal LASSO penalty parameter was determined using an internal 10-fold cross-validation on the training set to minimize the binomial deviance. Ultimately, this rigorous process retained 5 DL-derived features alongside Mean_HU and LAM for the Hybrid (COM) model.

#### 2.5.2 Model training and evaluation

To evaluate the diagnostic value of different feature sets, the ExtraTrees classifier was selected for its robust performance. Model hyperparameters were optimized via a grid search nested within a 5-fold cross-validation framework strictly on the training dataset. The optimal ExtraTrees hyperparameters were determined as follows: n_estimators=132, max_depth=6, min_samples_split=2 and random_state=0. To address the class imbalance between T2DM and non-T2DM instances, class weights were automatically balanced inversely proportional to class frequencies during model training. Furthermore, the decision threshold for binary classification was explicitly set to 0.9 to optimize the model’s performance metrics. Based on this classifier, three distinct detective models were developed (1): a Baseline (Muscle) Model utilizing only the conventional explainable biomarkers; (2) a DL Model utilizing only the selected DL features; and (3) a Hybrid (COM) Model integrating both feature sets. To address potential confounding effects of patient demographics, a Clinical Baseline Model (incorporating age, gender, BMI, smoking, and drinking status) and an Integrated Model (combining the clinical baseline variables with the optimal COM imaging features) were additionally developed. The incremental diagnostic value of the imaging features over conventional clinical information was quantitatively evaluated in the external validation cohort by comparing the Area Under the Curve (AUC) values using the DeLong test. Model performance was subsequently evaluated on the independent internal and external validation datasets. Receiver operating characteristic (ROC) curves and the AUC were utilized to compare diagnotics performance. The DeLong test was utilized to quantitatively compare AUCs, with exact P-values and 95% confidence intervals (CIs) reported.

### 2.6 Statistical analysis

All analyses were conducted using Python (version 3.10). The Mann-Whitney U test and Chi-square test were applied to continuous and categorical variables, respectively. Feature selection was performed using the LASSO algorithm. Decision curve analysis (DCA) was applied to assess the clinical utility of the models, while calibration curves were used to evaluate detection accuracy. To interpret the decision-making process of the optimal Hybrid (COM) model and quantify individual feature contributions, SHAP analysis was performed. Specifically, SHAP summary and dependence plots were generated using the external validation dataset to ensure the generalizability of feature importance and to provide clinical transparency. Model performance was compared using the DeLong test, with statistical significance set at p < 0.05. The performance metrics included accuracy (ACC), specificity (SPE), sensitivity (SEN), positive predictive value (PPV), and negative predictive value (NPV). This study was conducted in accordance with the Declaration of Helsinki and approved by the Ethics Committee of Center I and Center II (Approval No. 2023LCSY082). The requirement for informed consent was waived by the Institutional Review Board.

## 3 Results

### 3.1 Patient characteristics

The baseline clinical characteristics of the patients are summarized in **Table 1**. A total of 1,209 patients were included, with ages ranging from 45 to 70 years (mean age, 57.56 ± 12.68 years). Participants with T2DM in both the training and internal validation cohorts were older than those without T2DM, whereas no significant gender differences were observed between groups.

**Table 1:** Clinical Characteristics of Patients in training, internal validation and external validation dataset.

Characteristics	Training dataset			Internal validation dataset			External validation dataset		
	Non-T2DM	T2DM	*p*	Non-T2DM	T2DM	*p*	Non-T2DM	T2DM	*p*
N	594	261		148	66		95	45	
Age	56.85±12.86	59.07±13.33	0.014	55.09±12.94	59.78±12.67	0.008	54.15±7.03	55.19±9.43	0.463
Gender, male(female)	290(304)	134(127)	0.497	75(73)	34(30)	0.743	50(45)	23(22)	0.866

T2DM type 2 diabetes mellitus, Non-T2DM non-type 2 diabetes mellitus.

### 3.2 MedSAM segmentation

The semi-automated segmentation workflow demonstrated high efficiency and reproducibility. In the randomly selected subset of 100 cases, the inter-observer agreement between the two radiologists was excellent, yielding an ICC of 0.89. The fine-tuned MedSAM model achieved an average Dice coefficient of 0.95 ± 0.09 and IoU of 0.95 ± 0.07 in the internal dataset. Importantly, the segmentation maintained robust performance in the external dataset cohort, achieving a Dice coefficient of 0.87 ± 0.10 and an IoU of 0.86 ± 0.07, confirming its generalizability across different centers.

### 3.3 Diagnostic model performance and comparison

Based on the robust ExtraTrees classifier and features extracted via the ResNet152 architecture, three detective models (Muscle, DL, and COM) were developed and compared (**Table 2** and **Figure 3**). In the external validation dataset, the optimal COM model achieved the highest AUC of 0.746 (95% CI: 0.675–0.817), an ACC of 0.697, a SEN of 0.734, and a SPE of 0.655. The baseline Muscle model yielded a substantially lower AUC of 0.515 (95% CI: 0.429–0.600) in the external validation set, while the DL model achieved an AUC of 0.722(95% CI: 0.647–0.797). Similar trends of superiority were observed in the training (AUC: 0.830, 95% CI: 0.890-0.941) and internal validation (AUC: 0.759, 95% CI: 0.713-0.841) datasets for the COM model. DeLong tests revealed that the COM model yielded a statistically significant improvement in AUC compared to the baseline Muscle model in both the internal (AUC 0.759 vs. 0.637, P = 0.011) and external validation datasets (AUC 0.746 vs. 0.515, P < 0.001). However, no statistically significant difference was observed between the DL model and the COM model across these two datasets (internal validation set: P = 0.264; external validation set: P = 0.161) (**Figure 4**). This statistically confirms the substantial incremental diagnostic value of integrating deep learning features with conventional metrics. Furthermore, the calibration curves exhibited good agreement between the predicted probabilities and actual observations for the DL and COM model across all cohorts (**Figure 5**).

**Table 2:** Performances of the diagnostic models in the study sets.

Model name	Dataset	ACC	AUC	95% CI	SEN	SPE	PPV	NPV
COM	Training	0.749	0.83	0.790 - 0.868	0.727	0.772	0.776	0.723
COM	Internal validation	0.729	0.759	0.693 - 0.825	0.743	0.713	0.743	0.713
COM	External validation	0.697	0.746	0.675 - 0.817	0.734	0.655	0.704	0.687
DL	Training	0.832	0.916	0.890 - 0.941	0.829	0.836	0.846	0.819
DL	Internal validation	0.738	0.777	0.713 - 0.841	0.735	0.743	0.761	0.714
DL	External validation	0.680	0.722	0.647 - 0.797	0.606	0.762	0.740	0.634
Muscle	Training	0.741	0.824	0.784 - 0.864	0.649	0.841	0.816	0.688
Muscle	Internal validation	0.636	0.637	0.562 - 0.711	0.991	0.238	0.593	0.960
Muscle	External validation	0.545	0.515	0.429 - 0.600	0.681	0.393	0.557	0.524

AUC area under the curve, ACC accuracy, SEN sensitivity, SPE specificity, 95% CI confidence interval = 95%.

**Figure 3:**
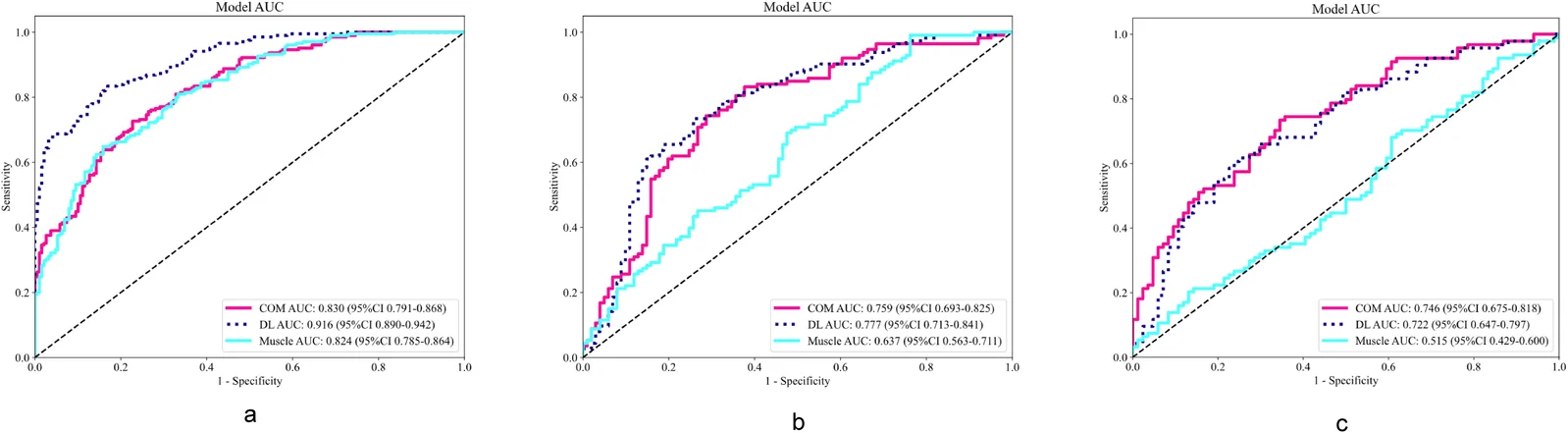
The ROC curves in the study sets. **(a-c)** represent the ROC curves for the models in the training dataset **(a)**, internal validation dataset **(b)**, and external validation dataset **(c)**, respectively.

**Figure 4:**
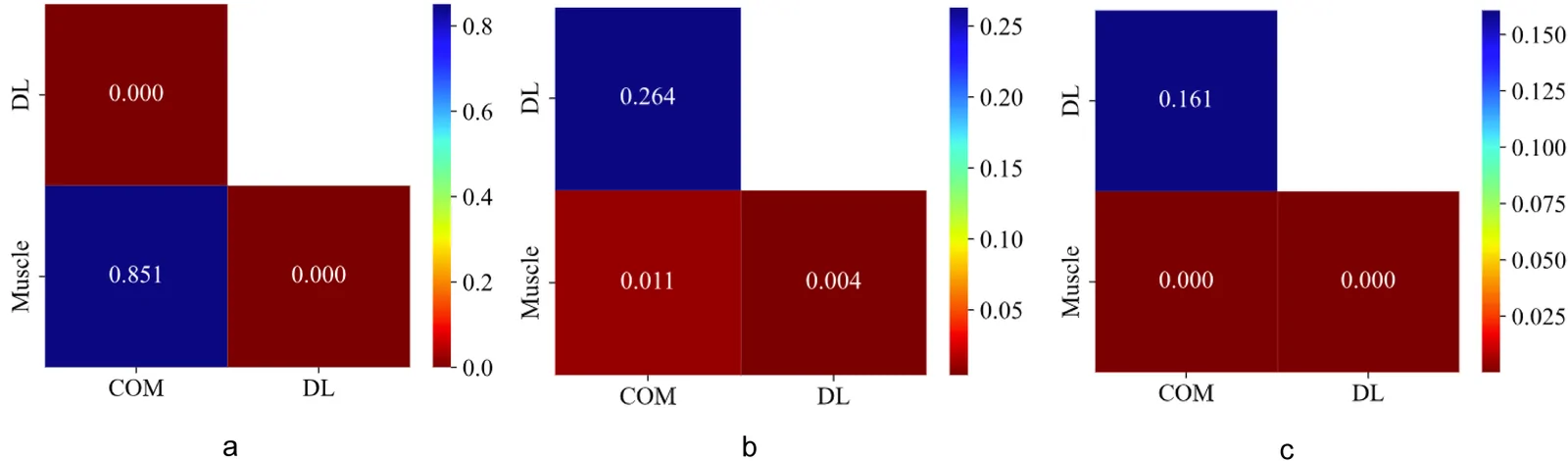
The DeLong tests in the study sets. **(a-c)** represent the DeLong tests between the models in the training dataset **(a)**, internal validation dataset **(b)**, and external validation dataset **(c)**, respectively.

**Figure 5:**
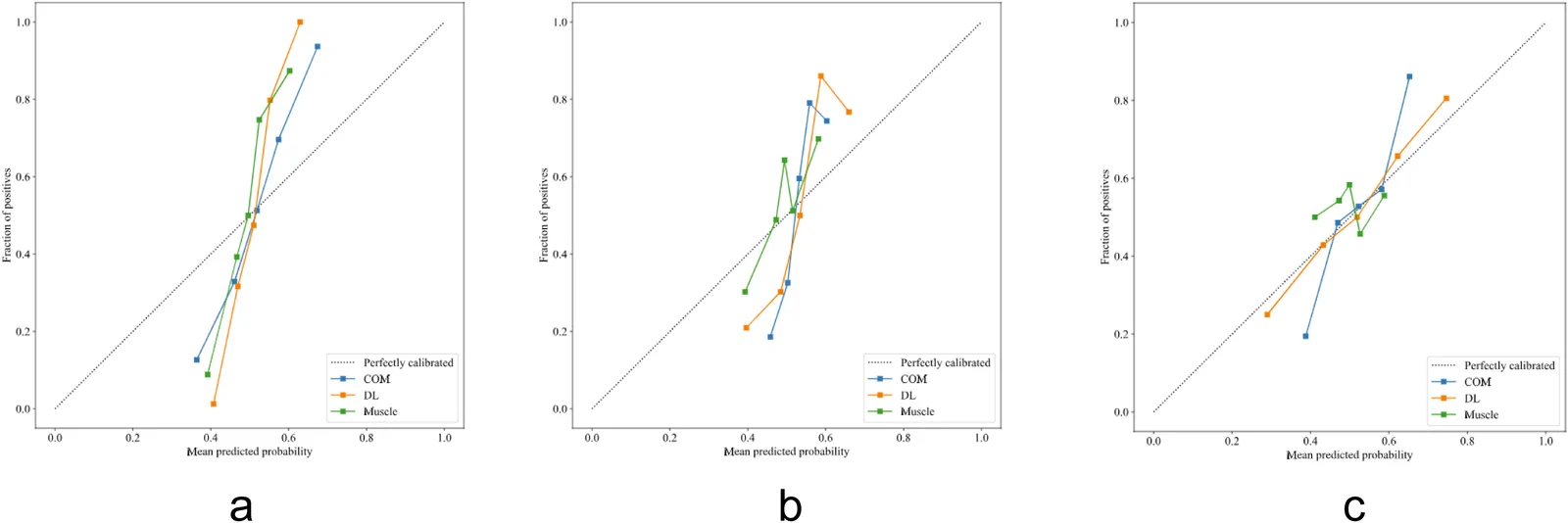
The calibration curves in the study sets. **(a-c)** represent the calibration curves for the models in the training dataset **(a)**, internal validation dataset **(b)**, and external validation dataset **(c)**, respectively. The black diagonal line represents the ideal perfect calibration.

### 3.4 Feature interpretability and clinical transparency

To elucidate the decision-making process of the optimal COM model and prevent “black-box” detections, SHAP analysis was conducted on the external validation dataset. As illustrated in the SHAP dependence plots (**Figure 6**), both the conventional biomarker (Mean_HU) and the deep learning-derived features (e.g., DL_0, DL_3) emerged as strong, independent detectors. Specifically, a lower Mean_HU (indicating lower muscle attenuation) was consistently associated with a higher SHAP value, signifying an increased predicted risk of T2DM. This observation is consistent with established clinical knowledge. Concurrently, the selected DL features exhibited robust, monotonic correlations with the model output, demonstrating that these radiomic signatures capture additive, sub-visual structural variations beyond macroscopic muscle density.

**Figure 6:**
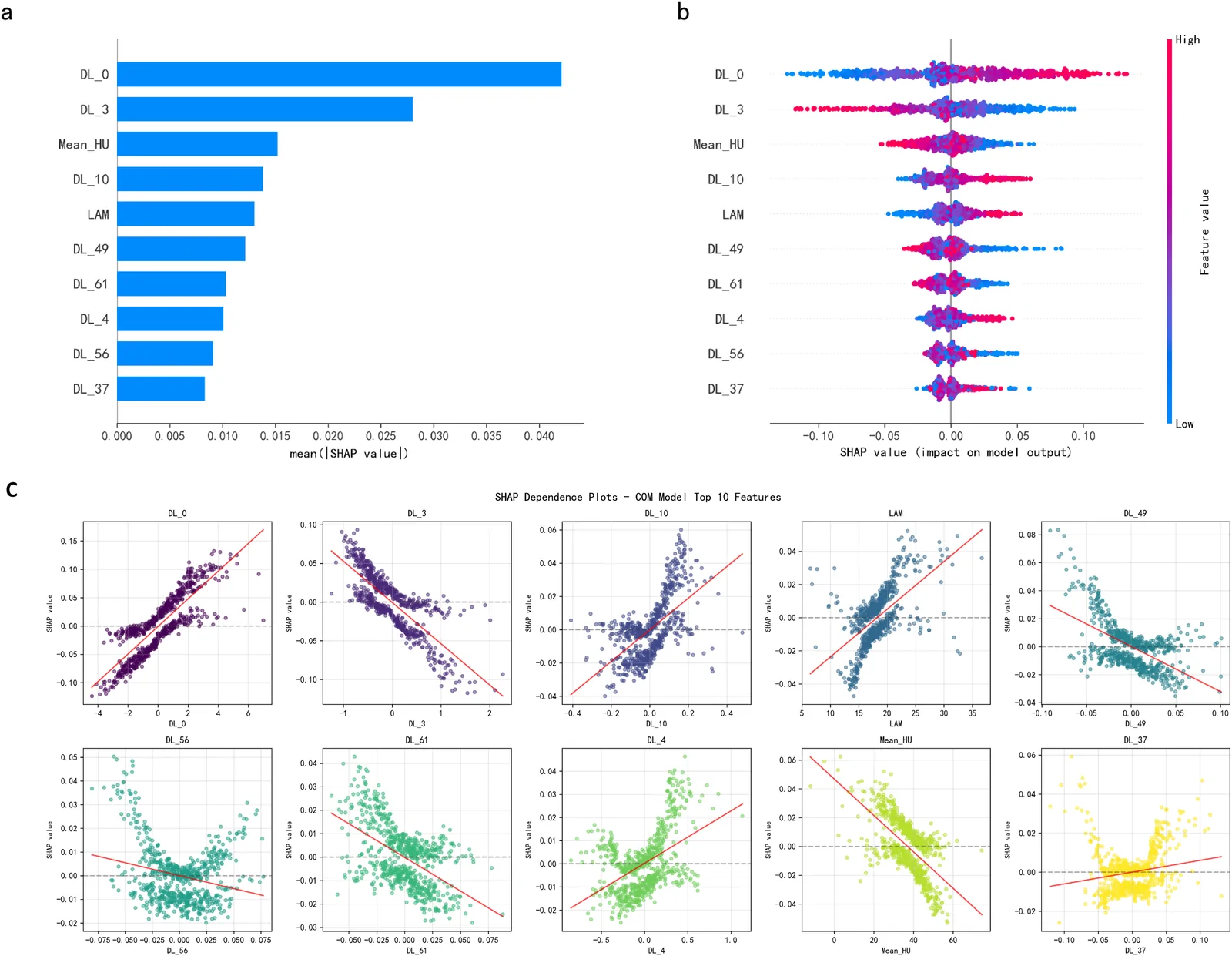
SHAP analysis of the optimal COM (Hybrid) model in the external validation dataset. > **(a)** SHAP summary plot illustrating overall feature importance and distribution. Features are ranked on the y-axis by their mean absolute SHAP values. Dot color represents the actual feature value (blue = low, pink/red = high), and the x-axis indicates the impact on predicting T2DM. **(b, c)** SHAP dependence plots detailing the relationship between actual measurement values (x-axis) and SHAP values (y-axis) for the top features. A SHAP value > 0.0 indicates an increased predicted risk of T2DM. Red lines represent linear regression trends.

### 3.5 Incremental diagnostic value over clinical confounders

To determine whether the deep learning-derived features merely captured age- or adiposity-related anatomical changes rather than diabetes-specific abnormalities, we evaluated their incremental diagnostic value against clinical confounders in the external validation cohort (**Table 3**) (Similar evaluations for the training and internal validation cohorts are detailed in **Supplementary Table 1**). The Clinical Baseline model performed poorly, with an AUC of 0.513. In contrast, the pure imaging COM model reached an AUC of 0.746, performing significantly better than the clinical variables alone (DeLong p < 0.001). Furthermore, adding clinical data to the imaging features (the Integrated model) resulted in an AUC of 0.735, which did not show any significant improvement over the pure COM model (DeLong p = 0.324).

**Table 3:** Diagnostic performance of the clinical, imaging, and integrated models in the external validation cohort.

Model name	Features included	ACC	AUC	95% CI	SEN	SPE	PPV	NPV	DeLong p-value
Clinical Baseline	Age, Gender, BMI, Smoking, Drinking	0.564	0.513	0.411-0.608	0.457	0.671	0.582	0.553	< 0.001
COM	Mean_HU, LAM, DL-features	0.697	0.746	0.675-0.817	0.734	0.655	0.704	0.687	Ref.
Integrated Model	Clinical + COM features	0.709	0.735	0.665-0.806	0.808	0.600	0.689	0.740	0.342

## 4 Discussion

This study proposes a novel, non-invasive strategy for opportunistic screening for T2DM using a hybrid deep imaging framework based on pectoralis muscle features extracted from LDCT scans in a health check-up population. Rather than relying solely on conventional macroscopic metrics, we demonstrated that integrating deep learning-derived features (via ResNet152) with simple explainable biomarkers (the COM model) significantly enhanced detection capability over the Muscle model. This hybrid approach achieved moderate but stable diagnostic performance across multi-center cohorts (training dataset AUC: 0.830; internal validation dataset AUC: 0.759; external validation dataset AUC: 0.746).

Skeletal muscle is not only the largest target organ for insulin but also plays a key role in glucose uptake. In T2DM, skeletal muscle metabolism undergoes significant alterations, including mitochondrial dysfunction and disruptions in amino acid metabolism (14, 15). These changes may contribute to both structural and compositional alterations in muscle, which can manifest as detectable imaging features on CT. Compared with conventional methods for body composition assessment, such as Dual-energy X-ray absorptiometry (DXA) or bioelectrical impedance analysis (BIA), CT offers higher spatial resolution for muscle quantification and architecture characterization (15). Leveraging LDCT for dual purposes—lung cancer screening and T2DM risk stratification—maximizes the clinical utility of routine imaging. Individuals identified as high-risk for T2DM can subsequently undergo confirmatory blood-based testing to enable early detection and timely intervention.

Accurate and reproducible quantification of the pectoralis muscle depends on reliable image segmentation. Manual ROI segmentation is time-consuming, subjective, and impractical for large-scale studies. To overcome this barrier, we employed MedSAM, a Transformer-based segmentation framework, which demonstrated excellent accuracy and efficiency. While CNN-based approaches such as ResNet and U-Net have shown good performance in pectoralis major muscle segmentation (16, 17), their generalizability is often limited (18, 19). MedSAM, by contrast, leverages the strengths of Transformer architecture to enhance contextual representation and adaptability to diverse anatomical variations. Our findings confirm that MedSAM enables efficient and accurate pectoralis segmentation, thereby establishing a reliable foundation for subsequent diagnostic analyses. DL has been increasingly applied in diabetes management, including prediction, diagnosis, complication screening, and biomarker discovery (20, 21). Recent studies have emphasized muscle imaging as a surrogate marker of metabolic health, such as the automated quantification of intramuscular fat content on whole-body MRI for diabetes risk prediction (22). While previous literature has established the diagnostic value of simple, macro-level muscle metrics (such as muscle area and attenuation) for T2DM (13, 23), our findings revealed that relying exclusively on these conventional biomarkers (the baseline Muscle model, AUC 0.515)yielded limited diagnostic accuracy. This may be because traditional indicators are susceptible to influences from scanners and population variations. To overcome this, we utilized MedSAM to automatically segment the pectoralis muscle and constructed a Hybrid (COM) model integrating both conventional explainable biomarkers (e.g., SMI, Mean_HU) and high-dimensional DL features extracted via the ResNet152 architecture. The COM model(AUC 0.746) successfully restored diagnostic stability and generalizability across diverse centers. Our study demonstrated that DL and COM model significantly outperformed the baseline Muscle model across all cohorts. This suggests that while macroscopic imaging characteristics serve as fundamental baseline indicators, deep learning networks are capable of capturing complex, sub-visual deep imaging and textural alterations within the muscle tissue. The integration of both provides substantial incremental diagnostic value, creating a more robust and comprehensive tool for opportunistic T2DM identification.

To address whether the imaging model simply reflects general aging or adiposity, we evaluated an Integrated Model. Interestingly, in external validation adding comprehensive clinical confounders to the COM model did not significantly improve its diagnostic performance (DeLong P = 0.324). This suggests that the extracted deep learning features already capture the relevant structural changes associated with T2DM. In a real-world workflow, the fact that clinical variables do not provide incremental value actually highlights the utility of the COM model. It can automatically stratify patient risk using only routine chest CT scans. This eliminates the need to acquire supplementary clinical records, making it highly suitable for large-scale opportunistic screening. To further validate the clinical transparency and biological grounding of our framework, we employed SHAP analysis to interpret the decision-making process of the optimal COM model. The SHAP dependence plots revealed that lower Mean_HU (muscle attenuation) was consistently associated with a higher risk of T2DM. This finding is highly consistent with the established physiological link between myosteatosis and insulin resistance (24). Furthermore, the high importance ranking of DL-derived features in the SHAP summary plot suggests that these signatures capture relevant structural variations that contribute to the model’s discriminative ability, independent of conventional metrics. Importantly, while SHAP provides robust statistical explainability regarding feature contributions to the model’s output, it does not prove direct biological causality or confirm underlying pathophysiology. Nevertheless, it offers supportive evidence that the model’s decision-making process is grounded in clinically relevant structural alterations rather than random noise. By visualizing these feature contributions, we bridge the gap between ‘black-box’ deep learning and clinical interpretability, providing a more reliable tool for opportunistic T2DM screening.

Our study has some limitations. Firstly, this was a cross-sectional study, limiting our ability to infer causal relationships. Prospective cohort studies are needed to establish temporal associations between pectoralis features and T2DM development. Secondly, external validation was performed using data from only one center, which restricts generalizability. Broader validation with multi-center datasets is necessary to improve model robustness. Additionally, the lack of detailed clinical information (e.g., medical history, waist circumference, lifestyle, physical activity, metabolic variables, comorbidities, and medication history, etc.) precluded a more comprehensive assessment of potential confounders. Future studies should integrate imaging, clinical, and physiological features to enhance diagnostic accuracy and interpretability.

In conclusion, our pure DL and COM models achieved moderate, comparable performance in identifying the presence of T2DM from routine LDCT. Retaining biological transparency, the hybrid model serves as a semi-automated, non-invasive, efficient, and zero-additional-cost tool for the opportunistic detection and concurrent triaging of unrecognised T2DM during lung cancer screening. Incorporating such transparent and biologically grounded imaging biomarkers into clinical workflows has the potential to facilitate personalized screening strategies and improve early detection of T2DM.

## Data Availability

The raw data supporting the conclusions of this article will be made available by the authors, without undue reservation.
