# Supplementary material for: Interpretable hybrid deep learning features of pectoralis on low-dose chest CT for detecting type 2 diabetes
Source: Front Endocrinol (Lausanne). 2026 Sep 16;17:1926903. doi: 10.3389/fendo.2026.1926903 (PMC13623598; doi:10.3389/fendo.2026.1926903)
Supplement: Supplementary file 1 [file Table1.docx]

Supplementary Table S1. Diagnostic performance of the Clinical, COM, and Integrated models in the study datasets.

| Model name | Dataset | ACC | AUC | 95% CI | SEN | SPE | PPV | NPV |
| --- | --- | --- | --- | --- | --- | --- | --- | --- |
| Clinical  Baseline | Training | 0.598 | 0.640 | 0.601-0.679 | 0.512 | 0.692 | 0.648 | 0.562 |
|  | Internal validation | 0.547 | 0.624 | 0.549-0.700 | 0.483 | 0.622 | 0.602 | 0.504 |
|  | External validation | 0.564 | 0.513 | 0.411-0.608 | 0.457 | 0.671 | 0.582 | 0.553 |
| COM | Training | 0.749 | 0.830 | 0.790 - 0.868 | 0.727 | 0.772 | 0.776 | 0.723 |
|  | Internal validation | 0.729 | 0.759 | 0.693 - 0.825 | 0.743 | 0.713 | 0.743 | 0.713 |
|  | External validation | 0.697 | 0.746 | 0.675-0.817 | 0.734 | 0.655 | 0.704 | 0.687 |
| Integrated  Model | Training | 0.732 | 0.792 | 0.763 - 0.820 | 0.723 | 0.742 | 0.759 | 0.705 |
|  | Internal validation | 0.715 | 0.740 | 0.673 - 0.806 | 0.885 | 0.525 | 0.676 | 0.803 |
|  | External validation | 0.709 | 0.735 | 0.665-0.806 | 0.808 | 0.6 | 0.689 | 0.740 |
